# A Contrastive Dual-Task Framework for Few-Shot Traffic Classification in IoT Networks

**DOI:** 10.3390/s26113471

**Published:** 2026-05-31

**Authors:** Zikui Lu, Mo Chen, Sailong Cui, Bingbing Zhao, Yaoyuan Zheng

**Affiliations:** College of Computer Science, Beijing Information Science and Technology University, Beijing 102206, China

**Keywords:** IoT, encrypted traffic classification, contrastive learning, few-shot learning

## Abstract

Classifying encrypted sensor traffic is critical for the security and management of Internet of Things networks, particularly in Mobile Edge Computing (MEC) environments. Existing methods often require extensive task-specific labeled data to adapt to emerging traffic categories and may also fail to distinguish intrinsic traffic behaviors from patterns introduced by shared communication libraries, which can degrade classification accuracy under distribution shifts. To address these issues, we propose CDTF, a contrastive dual-task framework for transferable and few-shot traffic representation learning. CDTF adopts a hybrid pre-training strategy that jointly optimizes supervised triplet pretraining (STP) and self-supervised dynamic burst masking (DBM). STP uses base-class labels as structural anchors to explicitly constrain distance relationships by aligning intra-class samples and separating inter-class samples, thereby mitigating interference from shared network components. DBM models global semantic structures and enhances the robustness of traffic representations against network noise and distribution shifts. By learning discriminative and contextual representations in a shared embedding space via these two tasks, CDTF can rapidly adapt to novel categories through lightweight fine-tuning, thereby substantially reducing the reliance on large-scale fine-grained supervision in downstream tasks. Experimental results across seven public and two custom datasets, across diverse environments, show that the proposed framework outperforms state-of-the-art methods. Under the few-shot setting, CDTF improves Precision by 4.61 percentage points over the strongest baseline, with statistical significance confirmed by a paired *t*-test (p<0.05).

## 1. Introduction

The widespread deployment of smart devices drives the explosive growth of encrypted network traffic within Internet of Things environments. Mobile Edge Computing (MEC) shifts traffic processing to the network edge to reduce end-to-end latency and alleviate bandwidth bottlenecks in backbone networks [1]. By enabling local anomaly filtering and fine-grained service classification, encrypted traffic analysis provides the foundation for edge network management and proactive defense systems. In this context, edge nodes must identify highly heterogeneous traffic at a fine granularity, distinguishing benign operational flows, such as video and sensor telemetry, from malicious activities, including anomaly probing and command-and-control communications [2,3]. These classification results enable dynamic bandwidth allocation and edge computing offloading, while directly informing critical operational decisions such as triggering anomaly alerts, enforcing access control, and isolating networks. Therefore, an accurate and efficient encrypted traffic classification mechanism is essential to ensure quality of service (QoS) in IoT systems and enhance network-edge security resilience.

However, analyzing encrypted traffic in IoT environments poses practical challenges. Specifically, different applications and services frequently invoke identical communication libraries, reuse similar cryptographic stacks, or route data through shared content delivery networks [4]. As a result, this sharing of underlying components causes diverse traffic behaviors to exhibit highly similar patterns in packet length, timing, and burst transmission. Consequently, classification methods risk misinterpreting this shared network noise as inherent class-discriminative features, leading to class confusion. Beyond these structural similarities, the continuous evolution of downstream data distributions complicates precise traffic classification. In particular, the constant emergence of novel smart devices, unknown application protocols, and malware variants drives a rapid expansion of traffic categories. While existing methods rely heavily on large, annotated datasets to address these new tasks, collecting extensive data for novel categories is cost-prohibitive and time-consuming. Furthermore, strict computational and storage constraints prevent edge nodes from sustaining the substantial overhead of frequent model retraining.

To reduce annotation costs and improve the transferability of traffic representations, recent studies have introduced pre-training paradigms into encrypted traffic analysis [5]. Representative methods, such as ET-BERT, TrafficFormer, and YaTC, learn general traffic representations through masked reconstruction, contextual modeling, or multi-level flow representation. Although these methods reduce the dependence of downstream tasks on large-scale labeled data, their pre-training objectives mainly focus on temporal dependencies within individual flows, local context, or token-level semantics, and provide limited modeling of inter-sample relationships and class-level structural constraints. In IoT environments, traffic from different categories often shares communication libraries, cryptographic stacks, or underlying network components. Objectives based only on single-sample reconstruction or context prediction may therefore preserve these shared transmission patterns, rather than separate the intrinsic behavioral features of different classes. As a result, existing pre-trained representations remain vulnerable to class confusion in few-shot and cross-distribution scenarios. Therefore, encrypted traffic representations should not only capture contextual semantics but also explicitly enhance inter-class separability, thereby improving their accuracy and transferability under novel categories and distribution shifts.

Based on these observations, we propose CDTF, a contrastive dual-task hybrid pre-training framework for encrypted traffic classification. CDTF integrates Supervised Triplet Pretraining (STP) and self-supervised Dynamic Burst Masking (DBM), enabling the model to jointly learn structured metric relationships and contextually robust representations. STP utilizes available base-class annotations to construct anchor-positive-negative triplets. Through metric constraints, it explicitly aligns intra-class samples and separates inter-class samples, thereby providing structured supervision for the embedding space and mitigating inter-class representation confusion. Meanwhile, DBM reconstructs masked tokens, encouraging the encoder to capture stable contextual dependencies and enhancing its robustness to network noise, local perturbations, and distribution shifts. Through this hybrid design, CDTF learns transferable representations that are both discriminative and robust from available base-class traffic, facilitating rapid adaptation to novel categories with limited task-specific labeled samples.

The contributions of this work are summarized as follows:

- We propose CDTF, a contrastive dual-task hybrid pre-training framework for encrypted traffic classification in IoT networks. CDTF introduces contrastive representation learning into hybrid pre-training by organizing traffic samples in a shared embedding space, where transferable representations can be learned from available base-class data and efficiently adapted to novel traffic categories with limited task-specific labeled samples.
- We design two pre-training tasks, namely Supervised Triplet Pretraining (STP) and Dynamic Burst Masking (DBM), to address inter-class representation confusion and contextual instability in encrypted traffic. STP constructs triplets from base-class annotations to explicitly enhance intra-class compactness and inter-class separability, thereby mitigating the influence of shared network components and common communication libraries. DBM reconstructs masked BURST tokens in a self-supervised manner to capture stable contextual dependencies and improve robustness against noise, local perturbations, and distribution shifts.
- Extensive experiments on seven public datasets and two custom datasets demonstrate that CDTF achieves state-of-the-art performance across multi-task, few-shot, and distribution-shift scenarios. CDTF improves Precision over the strongest baseline by 4.14 percentage points in website traffic classification, 4.61 percentage points under the few-shot setting, and 2.89 percentage points under cipher-suite distribution shift, with all gains statistically significant under paired *t*-tests (p<0.05).

The remainder of the paper is structured as follows: Section 2 reviews the related work, Section 3 introduces the proposed framework, Section 4 presents the experiments and analysis, and Section 5 concludes the paper.

## 2. Related Work

### 2.1. Feature-Based Traffic Classification

Traditional methods and machine learning approaches fundamentally rely on feature engineering for traffic classification. Early methods typically utilize metadata, such as port numbers, transport-layer protocols, five-tuples, and handshake fields, for classification. Although primarily used for protocol recognition, initial application classification, and Quality of Service (QoS) management, the reliability of these methods is severely compromised by dynamic port allocation and tunneling mechanisms [6]. In contrast, Deep Packet Inspection (DPI) identifies specific applications and protocols by matching application-layer features or protocol signatures within packet payloads. For instance, for P2P traffic identification, Sen et al. [7] extracted application-layer signatures and designed online filters, achieving precise classification by analyzing only a limited number of packets during the initial connection phase. nDPI combines packet header and payload information for protocol classification, proving effective on high-speed links [8]. While these methods are interpretable for identifying plaintext protocols and applications, their performance heavily relies on visible payloads and the maintenance of signature databases. Consequently, the widespread deployment of cryptographic protocols, such as TLS, QUIC, and VPNs, has severely limited the efficacy of these methods.

To reduce reliance on plaintext payloads, researchers have shifted toward statistical flow-based methods. These methods typically model traffic at the flow level, extracting a comprehensive set of statistical features from NetFlow or similar records. Specifically, these features include five-tuples, total packet and byte counts, flow durations, packet length distributions, and inter-packet arrival times. For example, AppScanner [9] classifies mobile applications using support vector machines and random forests based on packet length vectors. For the website fingerprinting identification, CUMUL [10] identifies websites by extracting fixed-dimensional features from cumulative packet length curves. Furthermore, BIND [11] classifies encrypted traffic by incorporating dependencies within bidirectional packet sequences. Anderson et al. [12] combined Markov transition probabilities of packet lengths with TLS handshake metadata to effectively classify malware traffic. HST [13] classifies application traffic by extracting statistical randomness features from payloads. Shamsimukhametov et al. [14] integrated unencrypted TLS handshake metadata with packet length and inter-packet arrival time features to achieve service classification.

Although feature-based methods support a variety of tasks, their efficacy is fundamentally limited by a heavy reliance on manually engineered features and static rules. These approaches struggle to capture the complex contextual semantics and inherent structural dependencies within network traffic. Moreover, the constant emergence of novel smart devices, unknown protocols, and malware variants severely degrades the generalization of fixed statistical features. As a result, these methods are increasingly inadequate for dynamic and continuously evolving network environments.

### 2.2. Deep Learning Methods

Deep learning methods, which automatically extract high-level features from encrypted flows, have become a mainstream approach for encrypted traffic classification [15]. Deep Fingerprinting (DF) [16], a CNN-based method, achieved website identification accuracy of more than 98% on undefended Tor traffic. FS-Net [17] is an end-to-end RNN-based model for encrypted traffic classification that learns representative features directly from raw flows. Although these methods have promoted the shift from manual feature engineering to automated representation learning, most of them still depend heavily on large-scale task-specific annotated data. In addition, they mainly model local patterns within individual flows or sessions.

To further exploit structural dependencies within network traffic, research has explored inter-flow correlations, host behavioral relationships, and graph-based modeling. For example, BLINC [18] profiles host behaviors to observe communication patterns for application identification. Additionally, FlowPrint [19] constructs mobile application fingerprints by discovering temporal correlations among destination-related features, thereby facilitating mobile application classification. GraphDApp [20] constructs byte-level traffic graphs based on pointwise mutual information to extract fine-grained representations for accurate classification. Although leveraging structural dependencies significantly benefits traffic classification, existing methods are fundamentally constrained by their reliance on predefined heuristic relationships, such as host behavior and destination correlations. This tight coupling to specific tasks and data distributions makes them highly vulnerable to distribution shifts caused by novel devices, emerging applications, and malware variants. In contrast, CDTF entirely bypasses the need for predefined heuristics. By optimizing triplet constraints on the base class to minimize intra-class distances and maximize inter-class distances, CDTF achieves superior flexibility and adaptability in dynamic environments.

Contrastive learning and metric learning have been employed to enhance the discriminability of traffic representations. Through contrastive pre-training, PASS [21] mitigates class imbalance, traffic homogeneity, and annotation dependency by pulling positive sample pairs closer and pushing negative pairs apart to yield robust feature representations. CLE-TFE [22] applies supervised contrastive learning to strengthen traffic representations, integrating graph augmentation and cross-layer multi-task learning to achieve hierarchical traffic classification. However, existing methods primarily focus on class boundaries, leaving them vulnerable to local perturbations, missing contexts, and distribution shifts in encrypted traffic. In response, CDTF jointly optimizes supervised triplet constraints and self-supervised DBM within a unified hybrid pre-training stage. By simultaneously enforcing structural discrimination and contextual robustness, CDTF substantially enhances representation transferability in few-shot and cross-distribution scenarios.

### 2.3. Pretraining-Based Methods

To reduce the dependence of downstream tasks on large-scale labeled data, pre-training paradigms have been increasingly introduced into encrypted traffic analysis. ET-BERT [23] pre-trains contextualized datagram representations on large-scale unlabeled traffic and then fine-tunes the model with a small amount of task-specific labeled data, improving performance across multiple encrypted traffic classification tasks. YaTC [24] adopts a masked autoencoder with multi-level flow representations and extracts features through packet-level and flow-level attention mechanisms for application classification. TrafficFormer [25] further explores an efficient pre-trained model for traffic to improve transferability across different traffic classification tasks. To mitigate traffic homogeneity and class imbalance in encrypted traffic classification, CETP [26] combines contrastive pre-training with multi-granularity representations. Concurrently, for few-shot traffic identification, EAPT [27] employs an adversarial pre-trained Transformer to extract robust features from limited unlabeled data.

These methods typically learn general traffic representations through masked reconstruction, contextual modeling, or hierarchical representation, thereby reducing the reliance on large-scale labeled data. However, they primarily focus on intra-flow reconstruction and context prediction, lacking sufficient modeling of inter-class structural constraints in the embedding space. To address this, CDTF introduces a hybrid pre-training framework. It explicitly enhances intra-class compactness and inter-class separability via supervised metric constraints, while simultaneously learning stable contextual dependencies and improving robustness against local perturbations through masked burst reconstruction. By jointly optimizing structural discrimination and contextual robustness, CDTF enables rapid adaptation to novel categories and distribution shifts with minimal task-specific annotations.

## 3. Methodology

**Figure 1:**
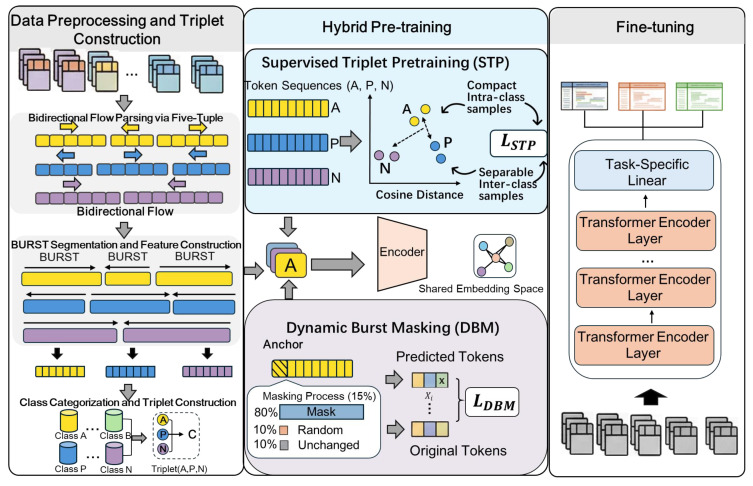
The overall architecture of CDTF. The framework comprises three main modules: (1) Data Preprocessing and Triplet Construction, which transforms raw packet traces into discrete BURST token sequences and constructs Anchor (A), Positive (P), and Negative (N) triplets using available base-class labels. (2) Hybrid Pre-training, which jointly optimizes supervised triplet pretraining (STP) and self-supervised dynamic burst masking (DBM). Specifically, STP imposes metric constraints based on cosine distance ($L_{\mathrm{STP}}$) to align intra-class samples and separate inter-class samples, while DBM masks anchor tokens and reconstructs them to capture contextual dependencies ($L_{\mathrm{DBM}}$). (3) Fine-tuning, where the shared Transformer encoder is combined with a task-specific linear classifier for efficient adaptation to downstream traffic classification tasks with limited labeled samples.

Figure 1 presents the overall architecture of CDTF. The framework consists of three stages: data preprocessing and triplet construction, hybrid pre-training, and fine-tuning. In the preprocessing stage, raw traffic is transformed into a discrete token representation suitable for sequence modeling. In the hybrid pre-training stage, supervised triplet pretraining and self-supervised dynamic burst masking are jointly optimized to learn transferable and robust representations of encrypted traffic. STP uses available labels from base-class data to impose inter-sample metric constraints, whereas DBM reconstructs masked tokens. In the fine-tuning stage, the pre-trained encoder is adapted to downstream target tasks through a lightweight supervised scheme, thereby reducing the reliance on large-scale labeled data.

### 3.1. Data Preprocessing and Triplet Construction

In real-world network environments, traffic data typically exhibit substantial heterogeneity across applications, protocols, and services. This inherent complexity makes it difficult for models to learn stable and discriminative representations directly from raw traffic. To address this issue, the preprocessing pipeline of CDTF is organized into three stages.

**Bidirectional Flow Parsing via Five-Tuple**

The first stage converts raw packet traces into structured bidirectional flows. In network traffic analysis, each packet is characterized by its timestamp, IP addresses, ports, protocol type, and payload content. Based on these attributes, packets are first grouped by the standard five-tuple, and the forward and reverse packet streams associated with the same communication session are subsequently aggregated into a single bidirectional flow. This process reorganizes the originally unstructured packet stream into flow-level samples, where each flow represents a complete bidirectional communication session and serves as the basic unit for subsequent BURST segmentation. Formally, let ${\left\{p_{i}\right\}}_{i=1}^{N}$ denote the packet stream extracted from raw traffic, and the flow set is denoted by

(1) $$F=\{f_{1},f_{2},\ldots ,f_{M}\},$$

where each flow $f_{m}\in F$ consists of the packets associated with one bidirectional communication session.

**BURST Segmentation and Feature Construction**

Although bidirectional flows are more structured than raw traffic, a single flow may still contain many packets over a relatively long time interval. Using the entire flow as model input is computationally inefficient and may introduce considerable redundancy, since many packets contribute only limited additional semantic information. In practice, traffic behaviors are often reflected more clearly in short-term interactive patterns between the two communicating endpoints. Therefore, each bidirectional flow is further segmented into a sequence of BURSTs, where each BURST consists of consecutive packets transmitted in the same direction. This design preserves localized interaction semantics while reducing unnecessary long-range redundancy.

After BURST segmentation, the payload bytes within each BURST are concatenated in temporal order to form a continuous byte sequence. This sequence is then converted into a hexadecimal string representation, which provides a standardized textual form for subsequent tokenization. To obtain model-ready features, bigram tokenization is applied to the hexadecimal sequence such that every two adjacent hexadecimal characters form one token. In this way, each BURST is represented as an independent token sequence and used as the basic sample unit for subsequent triplet construction and contrastive learning. Formally, for a bidirectional flow f∈F, its BURST sequence is denoted by

(2) $$B\left(f\right)=\left\{B_{1},B_{2},\ldots ,B_{K_{f}}\right\},$$

where $K_{f}$ is the number of BURSTs in flow *f*. For each BURST $B_{k}$, let $x_{k}$ denote the concatenated payload byte sequence in temporal order. The corresponding Bigram token sequence is defined as

(3) $$z_{k}=\mathrm{Bigram}\left(\mathrm{Hex}(x_{k})\right)=\left(z_{k,1},z_{k,2},\ldots ,z_{k,L_{k}}\right),$$

where $L_{k}$ denotes the token length of the *k*-th BURST. Collecting all BURST-level samples extracted from the flow set yields the final BURST sample set for subsequent learning.

**Class Categorization and Triplet Construction**

After all BURST-level samples are obtained, the samples in the base-class pre-training data are categorized according to their available class labels, thereby forming independent class-specific corpora. These labels are used only to construct metric relationships among samples during STP. To support supervised metric pre-training, the final preprocessing step constructs triplets from these corpora. Each triplet consists of an anchor sample (A), a positive sample (P), and a negative sample (N). Specifically, the anchor and positive samples are randomly drawn from the same class, whereas the negative sample is randomly drawn from a different class.

### 3.2. Hybrid Pre-Training

In the hybrid pre-training phase, CDTF introduces two tasks, supervised triplet pretraining and dynamic burst masking, to maximize the utility of limited labeled data. Given a triplet (A,P,N), the token sequences of the three BURST samples are fed into a shared encoder to obtain their corresponding vector representations. The STP objective is imposed on these sample-level representations, while the DBM objective is applied only to the masked token sequence of the anchor sample *A*. In this way, the encoder is jointly optimized by metric supervision at the sample level and token reconstruction at the token level. Both tasks share the same encoder parameters and are trained end-to-end under a unified objective, enabling the model to capture both global discriminative structure and local contextual dependencies in encrypted traffic.

#### 3.2.1. Supervised Triplet Pretraining

This task explicitly constrains the distance relationships among samples, encouraging the model to learn compact intra-class representations and separable inter-class representations. Specifically, STP minimizes the distance between *A* and *P* while maximizing the distance between *A* and *N*. As illustrated in the center of Figure 1, this objective encourages BURST samples from the same class to remain close in the representation space, while pushing samples from different classes farther apart. In this way, CDTF learns discriminative representations for encrypted traffic classification. The loss of STP is defined by the triplet loss:

(4) $$L_{\mathrm{STP}}=\max\left(0,d\left(h_{A},h_{P}\right)-d\left(h_{A},h_{N}\right)+\alpha \right)$$

where $h_{A}$, $h_{P}$, and $h_{N}$ denote the vector representations of samples *A*, *P*, and *N*, respectively, produced by the encoder. The function d(·,·) denotes the distance metric in the representation space, and cosine distance is adopted in this work. The parameter α is a predefined margin that enforces a minimum separation between intra-class and inter-class distances. When this constraint is violated, the loss becomes positive, and the model is updated accordingly. By explicitly optimizing relative distances among triplet samples, STP provides direct supervision on class structure and encourages the encoder to produce representations that are more suitable for downstream classification under limited labeled data.

#### 3.2.2. Dynamic Burst Masking

DBM does not require class labels and is formulated as a self-supervised token reconstruction task. This task simulates token corruption in real network environments to improve robustness to noise and local information loss, thereby mitigating the challenges caused by distribution shift and traffic homogeneity. Since the quality and stability of the anchor sample *A* are critical to metric learning, DBM is applied only to the token sequence of the anchor sample. By masking the input sequence of *A*, the model is encouraged to learn more robust contextual representations for the most important reference sample in triplet learning. This auxiliary objective also prevents the encoder from relying excessively on a small number of local token patterns and instead promotes a broader understanding of sequential context.

Let $z_{A}=\left(z_{1},z_{2},\ldots ,z_{L_{A}}\right)$ denote the original token sequence of the anchor sample, and let ${\bar{z}}_{A}$ denote the corresponding masked sequence. During masking, tokens in the anchor sequence are selected with a probability of 15%. Among the selected tokens, 80% are replaced with the [MASK] token, 10% are replaced with random tokens, and the remaining 10% are left unchanged. Let *k* denote the number of selected positions. Based on the masked anchor sequence, CDTF is trained to recover the original tokens at these positions. The loss of DBM is defined as

(5) $$L_{\mathrm{DBM}}=-\sum_{i=1}^{k}\log P\left(z_{i}|{\bar{z}}_{A};\theta \right)$$

where θ denotes the trainable parameters of CDTF, ${\bar{z}}_{A}$ denotes the masked token sequence of the anchor sample *A*, and $z_{i}$ denotes the original token at the *i*-th masked position. The probability $P(z_{i}|{\bar{z}}_{A};\theta )$ is predicted by the encoder based on the contextual information in the masked sequence. Through this reconstruction task, the encoder is encouraged to preserve fine-grained token semantics and contextual dependencies, which complements the sample-level discrimination imposed by STP.

In summary, the final hybrid pre-training objective is defined as the sum of the two losses:

(6) $$L=L_{\mathrm{STP}}+L_{\mathrm{DBM}}$$

The STP objective provides supervised metric constraints, whereas the DBM objective enhances robustness and contextual modeling. Their joint optimization enables CDTF to learn representations that are both structurally discriminative and contextually robust, providing a stronger initialization for subsequent task-specific fine-tuning under limited supervision.

### 3.3. Fine-Tuning

After pre-training, the encoder of CDTF can generate discriminative representations of encrypted traffic. However, under specific downstream tasks, these pre-trained representations may not fully capture task-specific characteristics. Fine-tuning is therefore performed to further adapt the model to the target task. Compared with the pre-training phase, the fine-tuning stage adopts a simplified architecture. Specifically, the auxiliary heads used for the pre-training tasks are removed, and a new task-specific classifier is constructed according to the number of classes in the downstream task. This design preserves the representation ability acquired during pre-training while enabling efficient adaptation with limited labeled samples.

Given an input BURST sample *B*, its token sequence is first fed into the pre-trained encoder to obtain the corresponding representation h. Based on this representation, the classifier produces the probability distribution over downstream classes:

(7) $$\hat{y}=\mathrm{softmax}\left(Wh+b\right),$$

where *W* and *b* denote the parameters of the task-specific classifier, and $\hat{y}$ denotes the predicted class distribution. Let y denote the one-hot ground-truth label of the input sample. The fine-tuning objective is defined by the cross-entropy loss:

(8) $$L_{\mathrm{FT}}=-\sum_{c=1}^{C}y_{c}\log{\hat{y}}_{c},$$

where *C* is the number of classes, and $y_{c}$ and ${\hat{y}}_{c}$ denote the ground-truth label and predicted probability of the *c*-th class, respectively.

During fine-tuning, the encoder and classifier parameters are jointly optimized using a limited number of labeled task-specific samples. In this way, the pre-trained knowledge learned from large-scale pretext tasks is transferred to the downstream task, while the model is further adjusted to capture task-specific decision boundaries. By removing the auxiliary pre-training heads and introducing a classifier tailored to the downstream label space, this fine-tuning mechanism enables efficient adaptation, accelerates convergence, and reduces reliance on large-scale fine-grained labels in the target domain under few-shot conditions.

## 4. Experiment

### 4.1. Experimental Setup

In this section, we evaluate the performance of CDTF across diverse scenarios. To this end, we conduct a comprehensive series of experiments, encompassing multi-task evaluations, few-shot and distribution shift evaluations, complexity analyses, and ablation studies.

#### 4.1.1. Datasets

In our experiments, we use a total of seven public datasets and two custom datasets. Specifically, we construct the pre-training dataset using the NonVPN subset of CIC-ISCX2016 [28], CSTNET-TLS1.3 [23], and ToN-IoT [29]. We then evaluate the model on the VPN subset of CIC-ISCX2016, CIC-IoT2023 [30], VisQUIC [31], CipherSpectrum [32], CIC-AndMal2017 and the custom datasets *Group1* and *Group2*. The detailed statistics of these datasets are summarized in Table 1.

ISCX-VPN: Covers common application and VPN protocols. We use its NonVPN subset for pre-training and the VPN subset to evaluate application classification performance.

CIC-IoT2023: Contains 34 attack categories, used to evaluate the model under diverse attack scenarios.

VisQUIC: Comprises QUIC-encrypted traffic from 18 websites, used to evaluate website traffic classification.

CIC-AndMal2017: Consists of Android malware traffic across three scenarios: Adware (4 categories), Scareware (11 categories), and SMSmalware (11 categories). We use it to evaluate malware detection performance.

CipherSpectrum: Features ciphertext from four encryption scenarios (AES-128, AES-256, ChaChaPoly, and a mixed scenario), each containing the same 41 traffic classes. We use it to assess model robustness across different cipher suites.

CSTNET-TLS1.3: Contains TLS 1.3 encrypted traffic from 120 service categories, used for model pre-training.

ToN-IoT: Includes nine heterogeneous IoT attacks within its malicious subset, used for model pre-training.

Group 1 and Group 2: Used to evaluate robustness against distribution shifts, with *Group 1* used only for downstream fine-tuning and *Group 2* used as an unseen test set. While completely disjoint in fine-grained labels, they strictly align across three high-level semantic intents: interactive communication (*Group 1*: ICQ; *Group 2*: AIM, Hangouts), information theft and control (*Group 1*: Backdoor, Hijacking; *Group 2*: MITM, FakeTaoBao), and resource abuse and fraud (*Group 1*: DDoS; *Group 2*: Beanbot, FakeJobOffer).

#### 4.1.2. Evaluation Metrics

We evaluate and compare the performance of the methods using four typical metrics, including accuracy (ACC), precision (PRE), recall (REC), and F1 score, and the calculation methods of these metrics are as follows:

(9) $$ACC=\frac{TP+TN}{TP+TN+FP+FN}$$

(10) $$PRE=\frac{TP}{TP+FP}$$

(11) $$REC=\frac{TP}{TP+FN}$$

(12) $$F1=2\times \frac{PRE\times REC}{PRE+REC}$$

#### 4.1.3. Implementation

**Data preprocessing and augmentation.** During data preprocessing, we remove IP addresses and port numbers, as they lack semantic relevance to the payload and make only a limited contribution to generalized representation learning. For hybrid pre-training, we construct a corpus comprising 10 GB of public data, enforcing a strictly disjoint split from the evaluation datasets to prevent data leakage. Furthermore, we expand the original corpus to 20 GB for pre-training through data augmentation. Specifically, when constructing contrastive triplets, we pair the same anchor with different positive and negative samples to generate diverse training instances. Additionally, applying masks to different positions within the same token sequence in the DBM task further enhances the diversity of the training data. Finally, to mitigate class imbalance, we apply an upsampling strategy during the fine-tuning stage.

**Model architecture and configuration.** The CDTF architecture is built on a four-layer Transformer encoder, with a hidden dimension of 512 and 8 self-attention heads. Following the work [23], sequential index encoding is used to represent positional information. The vocabulary contains 65,535 entries, and any out-of-vocabulary token encountered is mapped to the special [UNK] token. The margin hyperparameter α is empirically set to 0.5.

**Training Settings and Baselines.** All experiments are conducted on NVIDIA Tesla V100 GPUs. During hybrid pre-training, the model is optimized with AdamW using a learning rate of $2\times 10^{-5}$ and a batch size of 2048. The pre-training process runs for 100,000 iterations and requires approximately 75 GPU hours. To reduce overfitting to noise, early stopping is applied based on the validation loss with a patience of 10 epochs [33]. For performance comparison, CDTF is evaluated against several representative methods, including AppScanner [9], CUMUL [10], GraphDApp [20], CLE-TFE [22], ET-BERT [23], and TrafficFormer [25]. These baselines cover representative machine learning and deep learning methods for traffic analysis. To ensure a fair comparison, all baseline methods are implemented using their official code or public repositories, fine-tuned on the same downstream fine-tuning data, and tested on the same evaluation data. During evaluation, except in the few-shot scenarios, each dataset is randomly split into training, validation, and test sets at a ratio of 7:1:2. This random splitting process is repeated five times, and the mean and standard deviation are reported.

### 4.2. Multi-Task Evaluations

To evaluate the performance of CDTF across different task scenarios, we consider application classification, attack classification, website traffic classification, and malware detection. As shown in Table 2, CDTF achieves the best mean performance across all tasks. Compared with the strongest baseline, CDTF improves PRE and REC by 0.97 and 0.16 percentage points on CIC-ISCX2016, by 3.40 and 3.19 percentage points on CIC-IoT2023, and by 4.14 and 0.52 percentage points on VisQUIC, respectively. The significance markers further show that several improvements, including PRE on CIC-ISCX2016, REC on CIC-IoT2023, and PRE on VisQUIC, are statistically significant under the paired *t*-test with p<0.05.

On CIC-AndMal2017, CDTF also achieves the highest mean performance across all malware detection scenarios. The improvement is most pronounced in the Adware scenario, where CDTF exceeds the strongest baseline by 8.84 percentage points in PRE and 6.16 percentage points in REC. In the SMSmalware scenario, CDTF yields a small PRE gain of 0.45 percentage points but improves REC by 2.55 percentage points, suggesting that the advantage of CDTF is more evident in reducing missed detections than in improving precision. In the Scareware scenario, CDTF improves PRE and REC by 2.48 and 1.42 percentage points, respectively.

CDTF shows low standard deviations across all tasks, with the largest value being 1.87. In contrast, some baselines show much larger variation. These results indicate that CDTF not only achieves strong mean performance but also maintains stable behavior across repeated runs. Such stability is important for data-limited or distribution-sensitive traffic classification scenarios, where performance can be strongly affected by data splits and class composition.

### 4.3. Few-Shot and Distribution Shift Evaluations

To evaluate the robustness of the proposed method under few-shot conditions, we conduct evaluations by partitioning the datasets into training, validation, and testing sets at a ratio of 1:1:8. Compared to the standard setting, reducing the labeled fine-tuning data to only 10% inevitably causes a performance drop across all models. AppScanner and CLE-TFE suffer severe performance degradation, indicating their heavy reliance on large-scale annotated data. Despite the extremely limited labeled data, Table 3 demonstrates that CDTF consistently outperforms all baseline methods across the three datasets. Specifically, CDTF achieves the highest precision and recall. On the CIC-IoT2023 dataset in particular, CDTF surpasses the strongest baseline TrafficFormer by 4.61 percentage points in precision. When compared to CLE-TFE, a contrastive-learning-based method, CDTF maintains a significant advantage across all performance metrics. This demonstrates that the proposed method extracts highly generalizable traffic semantic features, ensuring its adaptability in environments with limited labeled data.

To evaluate the generalization ability of CDTF under distribution shifts, we conduct a cross-cipher suite transfer experiment. Specifically, all methods are trained on AES-128 traffic and then directly evaluated on AES-256, ChaChaPoly, and Mix, with no training samples from these target suites. This setting simulates distribution shifts caused by changes in encryption schemes. As shown in Table 4, CDTF achieves the highest mean PRE and REC across all conditions. On AES-256, CDTF improves PRE and REC over the strongest baseline by 2.89 and 0.38 percentage points, respectively. On ChaChaPoly, CDTF obtains gains of 1.82 and 0.66 percentage points, respectively. Under the mixed cipher suite setting, CDTF improves PRE and REC by 2.00 and 2.59 percentage points, respectively. These results show that the hybrid pre-training design helps maintain both discriminability and robustness when the encryption scheme changes, enabling CDTF to learn traffic representations that transfer effectively across cipher suites.

To further evaluate the robustness of CDTF under cross-distribution conditions, we design tasks with data distribution shifts. Specifically, we utilize *Group 1* and *Group 2*, representing an extreme configuration in which the downstream fine-tuning and testing sets share no common fine-grained label categories while preserving semantic consistency in three high-level service intents. For comparison, we select TrafficFormer, which demonstrated the strongest baseline on average in Table 4, as the baseline method.

**Figure 2:**
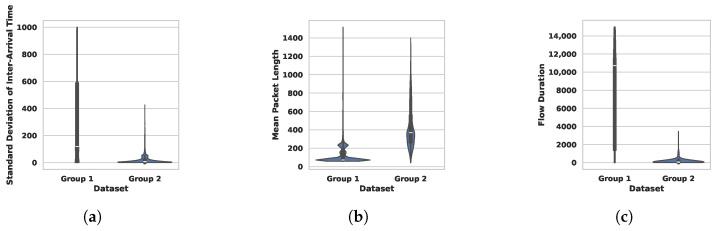
Feature distribution comparison between Group 1 and Group 2. (**a**) IAT standard deviation; (**b**) mean packet length; (**c**) flow duration.

**Figure 3:**
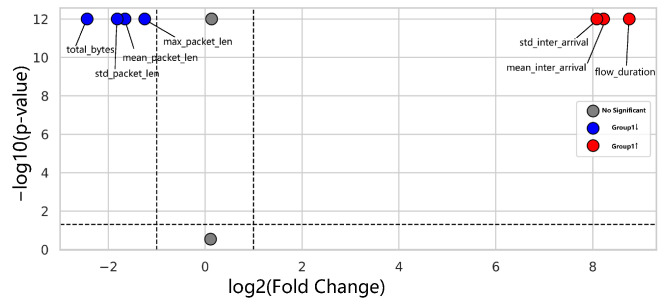
Volcano plot of feature significance between Group 1 and Group 2.

Figure 2a–c illustrate the distinct distribution patterns of the two groups. Notably, *Group 1* exhibits higher dispersion and more pronounced outliers in temporal features, whereas *Group 2* remains relatively compact. The volcano plot in Figure 3 further quantifies these disparities across various packet-level and temporal features. In this plot, the x-axis represents $\log_{2}\left(\mathrm{Fold}\;\mathrm{Change}\right)$, which indicates the magnitude and direction of the difference between the two groups, while the y-axis denotes $-\log_{10}\left(p\text{-}\mathrm{value}\right)$, reflecting statistical significance. Accordingly, points located in the upper-left and upper-right regions represent features characterized by both high statistical significance and substantial effect sizes. These observations confirm the existence of significant distributional shifts between the two groups.

In this setup, *Group 2* is equally partitioned into an adaptation set and a fixed testing set, with performance evaluated exclusively on the latter to ensure fairness. During downstream fine-tuning, data from the adaptation set is incrementally injected into *Group 1*. A 0% injection ratio represents a zero-shot scenario, where the classification of unseen traffic relies entirely on prior knowledge from *Group 1*. Conversely, a 100% injection implies full integration of the adaptation set.

**Figure 4:**
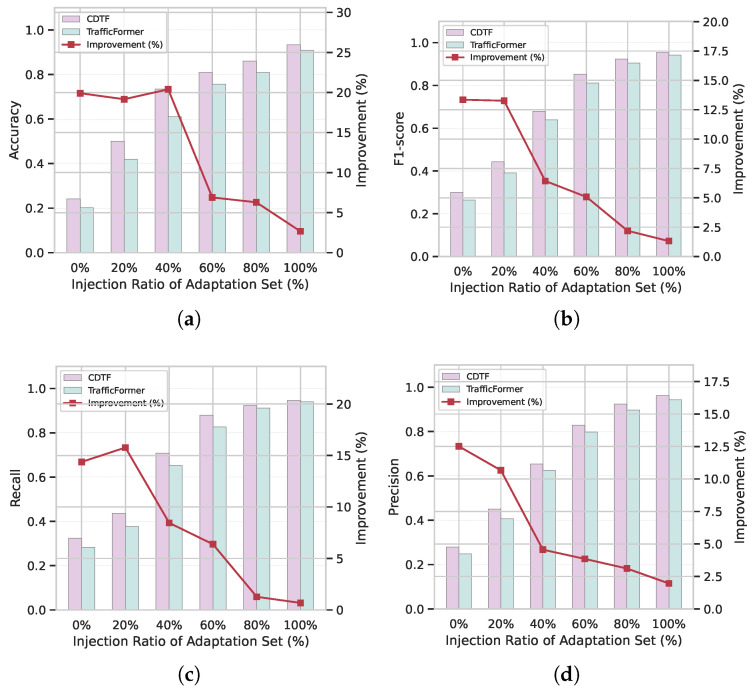
Performance comparison between CDTF and TrafficFormer under varying injection ratios of the adaptation set. (**a**) Accuracy under injection ratios; (**b**) F1-score under injection ratios; (**c**) Recall under injection ratios; (**d**) Precision under injection ratios.

Figure 4 illustrates the performance of CDTF and TrafficFormer across increasing injection ratios of target data. The results demonstrate that CDTF consistently outperforms TrafficFormer across all metrics. Notably, in the zero-shot scenario, CDTF exhibits robust generalization, achieving relative improvements of approximately 19.9% and 14.4% in ACC and REC, respectively, over the baseline. This confirms that CDTF effectively extracts the shared behavioral semantics of the high-level service intents, mitigating overfitting to the specific traffic families in *Group 1*. Furthermore, as the data-injection proportion increases, CDTF leverages the newly introduced task-specific data more efficiently. As evidenced by the improvement curves, CDTF rapidly adapts to novel fine-grained distributions, resulting in a pronounced performance gap at lower injection stages. Furthermore, it maintains a stable performance advantage even after the adaptation set is completely incorporated.

### 4.4. Complexity Analyses

As illustrated in Table 5, the proposed CDTF achieves a favorable balance between classification precision and memory efficiency, effectively alleviating constraints in resource-limited environments. While high-performance models such as ET-BERT and TrafficFormer require more than 500 MB of storage space, CDTF significantly reduces the memory footprint to 170 MB, which represents more than 60% memory savings. Despite this reduction in complexity, CDTF maintains superior performance compared to these methods.

Regarding temporal costs, CDTF demonstrates high efficiency during both the fine-tuning and deployment phases, fulfilling the requirements for rapid model iteration and real-time response. The fine-tuning process of CDTF requires only 92.58 s per 1000 samples, which is 66.1% faster than ET-BERT and 41.6% faster than TrafficFormer. Compared to CLE-TFE, which is also a contrastive learning-based method, the proposed approach reduces fine-tuning time by 80.1% and improves inference speed by approximately 6.8 times.

### 4.5. Ablation Study

We conduct an ablation study to verify the contribution of each component to the performance of our method on the CIC-ISCX2016 dataset. As shown in Table 6, removing the DBM or STP task results in a decline in F1 score by 29.42 percentage points and 41.27 percentage points, respectively, compared with the full framework. This indicates that the two pre-training tasks are complementary, each providing information for accurate traffic representation. Notably, the variant without STP exhibits a more significant performance drop, suggesting that supervised triplet pre-training plays a crucial role in modeling discriminative class relationships and enhancing inter-class separability in traffic representations.

Furthermore, the model trained without hybrid pre-training experiences a substantial performance drop of 40.12 percentage points in accuracy, 52.01 percentage points in precision, 46.86 percentage points in recall, and 49.34 percentage points in F1 score. These results demonstrate that our hybrid pre-training strategy is essential for learning generalized and transferable feature representations, which significantly enhance the downstream classification capability of the model.

## 5. Conclusions

In this paper, we propose CDTF, a contrastive dual-task hybrid pre-training framework for encrypted traffic classification in IoT networks. Beyond modeling contextual patterns within individual traffic samples, CDTF introduces supervised triplet pretraining to explicitly constrain inter-sample metric relationships, thereby enhancing intra-class compactness and inter-class separability under shared communication libraries and network components. Meanwhile, dynamic burst masking further improves contextual robustness by reconstructing masked BURST tokens in a self-supervised manner. Through the joint optimization of STP and DBM, CDTF learns transferable traffic representations that can be efficiently adapted to downstream tasks with limited labeled samples. Extensive experiments on seven public datasets and two custom datasets demonstrate that CDTF achieves state-of-the-art performance across multi-task, few-shot, and distribution-shift scenarios, with statistically significant gains over strong baselines. However, the current pre-training stage still relies on ground-truth base-class labels to construct supervised contrastive objectives. In future work, we will explore alternative class-level signals for reducing manual annotation requirements and further investigate the deployment and continuous optimization of CDTF on real resource-constrained edge nodes to better support practical MEC environments.

## Figures and Tables

**Table 1:** Statistics of the datasets used in the experiments.

Stage	Task	Dataset	Flow	Class
Hybrid Pre-training	-	CIC-ISCX2016 (NonVPN)	40 k	10
		ToN-IoT	27 k	9
		CSTNET-TLS1.3	54 k	120
Fine-tuning	Application Classification	CIC-ISCX2016 (VPN)	22 k	11
	Attack Classification	CIC-IoT2023	60 k	34
	Website Traffic Classification	VisQUIC	46 k	18
	Malware Detection	CIC-AndMal2017	100 k	4/11/11
		CipherSpectrum	41 k	41
	Distribution Shift	*Group1*	12 k	3
		*Group2*	13 k	3

**Table 2:** Comparison of multi-task evaluation performance across different methods.

**Task**	**Application Classification**		**Attack Classification**		**Website Traffic Classification**	
**Dataset**	CIC-ISCX2016		CIC-IoT2023		VisQUIC	
**Method**	PRE	REC	PRE	REC	PRE	REC
AppScanner	74.98±1.50	81.72±8.19	54.27±3.61	55.36±2.71	80.25±1.14	86.88±4.17
CUMUL	62.61±3.95	59.49±2.93	51.38±1.70	49.63±1.32	71.56±2.25	68.71±1.13
GraphDApp	49.82±10.22	39.25±13.45	33.15±1.84	42.31±2.02	73.92±2.20	70.49±3.22
ET-BERT	98.05±0.61	98.68±0.49	76.60±1.37	79.19±4.12	81.49±3.66	78.23±2.37
TrafficFormer	98.85±0.09	98.85±0.09	85.21±3.20	86.06±1.58	80.73±3.85	79.28±3.43
CLE-TFE	73.52±18.84	72.72±19.17	42.42±0.83	42.43±2.27	77.84±0.79	79.39±0.45
CDTF	${99.82\pm 0.04\;}^{†}$	99.01±0.26	88.61±1.13	${89.25\pm 0.80\;}^{†}$	${85.63\pm 0.25\;}^{†}$	87.40±0.11
**Task**	**Malware Detection**					
**Dataset**	CIC-AndMal2017 (Adware)		CIC-AndMal2017 (SMSmalware)		CIC-AndMal2017 (Scareware)	
**Method**	PRE	REC	PRE	REC	PRE	REC
AppScanner	50.43±0.78	47.97±0.60	67.10±4.30	66.60±3.32	60.97±5.98	54.01±5.84
CUMUL	43.37±0.13	43.03±0.10	47.48±1.01	47.56±2.04	19.70±0.80	19.29±0.81
GraphDApp	45.45±0.40	44.73±0.34	49.49±0.99	49.68±0.27	60.60±0.76	59.20±0.77
ET-BERT	70.06±4.46	71.42±2.82	77.21±3.82	76.30±2.11	74.68±1.54	70.42±3.31
TrafficFormer	66.24±10.25	68.87±5.37	75.28±7.79	74.07±4.33	64.32±1.18	69.67±8.66
CLE-TFE	55.56±6.46	56.10±1.06	63.20±4.91	68.95±1.98	61.84±7.87	58.39±1.95
CDTF	${78.90\pm 1.24\;}^{†}$	${77.58\pm 0.50\;}^{†}$	77.66±0.05	${78.85\pm 0.63\;}^{†}$	${77.16\pm 1.00\;}^{†}$	71.84±1.87

Note: All results are reported as mean ± standard deviation over five times. Bold results indicate the best performance, and ^†^ indicates that CDTF significantly outperforms the strongest baseline in the same column under a paired *t*-test with p<0.05.

**Table 3:** Performance comparison under few-shot conditions across different datasets.

Dataset	CIC-ISCX2016		CIC-IoT2023		VisQUIC	
Method	PRE	REC	PRE	REC	PRE	REC
AppScanner	47.78±12.07	38.50±3.40	33.15±1.10	33.88±2.35	64.77±1.03	64.70±0.38
CUMUL	29.02±3.27	29.70±2.07	21.74±1.11	24.63±0.09	51.41±0.76	51.98±0.73
GraphDApp	30.99±2.70	31.46±3.15	24.23±3.10	23.47±1.66	54.85±1.60	53.87±1.98
CLE-TFE	57.87±2.90	50.20±1.00	26.14±0.30	22.18±1.36	48.71±6.92	46.27±3.82
ET-BERT	84.27±0.29	83.17±0.26	63.08±0.46	62.35±0.62	69.26±1.08	69.10±0.77
TrafficFormer	83.36±0.06	82.68±0.12	64.55±0.36	63.39±1.17	74.11±0.42	72.77±0.37
CDTF	${85.44\pm 0.50\;}^{†}$	${84.18\pm 1.23\;}^{†}$	${69.16\pm 1.44\;}^{†}$	63.75±0.53	${75.85\pm 0.85\;}^{†}$	${74.46\pm 1.13\;}^{†}$

Note: All results are reported as mean ± standard deviation over five times. Bold results indicate the best performance, and ^†^ indicates that CDTF significantly outperforms the strongest baseline in the same column under a paired *t*-test with p<0.05.

**Table 4:** Performance evaluation under different cipher suites.

Method	AES-256		ChaChaPoly		Mix	
	PRE	REC	PRE	REC	PRE	REC
AppScanner	61.29±0.56	66.60±0.93	71.86±0.18	70.36±0.24	73.97±0.32	72.25±0.36
CUMUL	66.00±1.03	63.64±0.34	63.67±0.31	61.84±0.35	54.56±0.06	54.78±0.25
GraphDApp	53.33±0.06	52.63±0.50	67.26±0.05	65.28±0.29	62.60±0.51	63.28±0.08
CLE-TFE	65.09±1.55	66.91±0.97	72.61±0.03	73.95±1.77	68.59±0.47	67.14±0.89
ET-BERT	59.37±4.81	56.41±1.95	62.82±0.38	65.23±0.26	60.88±2.01	62.22±1.69
TrafficFormer	72.92±0.14	71.87±0.02	73.86±0.47	70.44±0.38	72.40±0.19	71.05±0.11
CDTF	${75.81\pm 0.49\;}^{†}$	${72.25\pm 0.33\;}^{†}$	${75.68\pm 0.66\;}^{†}$	74.61±0.38	${75.97\pm 0.56\;}^{†}$	${74.84\pm 0.38\;}^{†}$

Note: All results are reported as mean ± standard deviation over five times. Bold results indicate the best performance, and ^†^ indicates that CDTF significantly outperforms the strongest baseline in the same column under a paired *t*-test with p<0.05.

**Table 5:** Efficiency and performance comparison across different tasks.

Method	Model Size (MB)	Fine-Tuning Time (s)	Inference Time (s)	Performance			
				AC	TC	WTC	MD
AppScanner	8	109.18	0.45	74.98±1.50	54.27±3.61	80.25±1.14	50.43±0.78
CUMUL	3	96.21	0.12	62.61±3.95	51.38±1.70	71.56±2.25	43.37±0.13
GraphDApp	15	40.45	1.07	49.82±10.22	33.15±1.84	73.92±2.20	45.45±0.40
CLE-TFE	200	464.99	30.59	73.52±18.84	42.42±0.83	77.84±0.79	55.56±6.46
ET-BERT	504	272.92	14.83	98.05±0.61	76.60±1.37	81.49±3.66	70.06±4.46
TrafficFormer	506	158.61	7.39	98.85±0.09	85.21±3.20	80.73±3.85	66.24±10.25
CDTF	170	92.58	4.47	${99.82\pm 0.04\;}^{†}$	88.61±1.13	${85.63\pm 0.25\;}^{†}$	${78.90\pm 1.24\;}^{†}$

*Notes:* Fine-tuning time refers to the fine-tuning stage, and both fine-tuning time and inference time denote the time required to process 1000 data samples. Performance is reported in terms of the Precision metric. AC: Application Classification; TC: Attack Classification; WTC: Website Traffic Classification; MD: Malware Detection, evaluated under the Adware scenario. The ^†^ indicates that CDTF significantly outperforms the strongest baseline in the same column under a paired *t*-test with p<0.05.

**Table 6:** Ablation study of CDTF.

Method	Metric			
	ACC	PRE	REC	F1
w/o DBM	75.22 ± 1.33	69.98 ± 0.95	70.02 ± 1.88	69.91 ± 0.52
w/o STP	60.25 ± 0.48	59.10 ± 0.32	57.77 ± 0.52	58.06 ± 0.83
w/o hybrid pre-training	59.26 ± 2.25	47.81 ± 1.63	52.15 ± 1.04	49.99 ± 1.54
full CDTF	**99.38 ± 0.14**	**99.82 ± 0.04**	**99.01 ± 0.26**	**99.33 ± 0.10**

## Data Availability

The data presented in this study are available on request from the corresponding authors.
